# TOOKAD^®^ Soluble focal therapy: pooled analysis of three phase II studies assessing the minimally invasive ablation of localized prostate cancer

**DOI:** 10.1007/s00345-015-1505-8

**Published:** 2015-02-25

**Authors:** A. R. Azzouzi, E. Barret, J. Bennet, C. Moore, S. Taneja, G. Muir, A. Villers, J. Coleman, C. Allen, A. Scherz, M. Emberton

**Affiliations:** 1 Urology Department, University Hospital, CHU Angers, 4 rue Larrey, 49933 Angers Cedex 9, France; 2 Institut Mutualiste Montsouris (IMM), Paris, France; 3 Midtown Urology, Atlanta, GA USA; 4 University College London Hospital, London, UK; 5 NYU Langone Medical Center, New York, NY USA; 6 Kings College Hospital, London, UK; 7 University Hospital of Lille, Lille, France; 8 Memorial Sloan-Kettering Cancer Center, New York, NY USA; 9 Plant Sciences, Weizmann Institute of Science, Rehovot, Israel

**Keywords:** Localized prostate cancer, Focal therapy, WST11 TOOKAD^®^ Soluble, Vascular-targeted photodynamic therapy

## Abstract

**Purpose:**

To evaluate the 6-month effects of the recommended drug and light dosage in focal vascular-targeted photodynamic therapy (VTP) using TOOKAD^®^ Soluble in patients with localized prostate cancer (LPCa).

**Methods:**

We performed a pooled analysis of 117 men with LPCa, PSA <10 ng/mL, and Gleason score ≤7 (3 + 4), from 3 studies who received a 10-min intravenous infusion of a single dose of 4 mg/kg TOOKAD^®^ Soluble, activated by a 753-nm light at 200 J/cm delivered in the prostate by transperineal fibres under transrectal ultrasound guidance. Primary endpoint was 6-month negative biopsies in the treated lobe(s). PSA was measured at month 1, 3, and 6. Magnetic resonance imaging was performed at day 7, month 3, and 6. International Prostate Symptom Score (IPSS), International Index of Erectile Function (IIEF-5) and adverse events were reported at day 7, month 1, 3, and 6.

**Results:**

Month 6 negative biopsy rate was 68.4 % in the overall evaluable population (*N* = 114) and 80.6 % for patients treated by hemiablation with light density index (LDI) ≥ 1 (*N* = 67). Mean prostate necroses at week-1 were 76.5 and 86.3 %, respectively. In both groups, PSA levels at month 6 decreased by 2.0 ng/mL. Small changes from baseline for IPSS and IIEF-5 indicated a slight improvement in urinary function and a slight deterioration in sexual function.

**Conclusions:**

Focal VTP treatment with TOOKAD^®^ Soluble at 4 mg/kg and 200 J/cm resulted in a negative 6-month biopsy rate of 68.4 % for the whole population and 80.6 % for patients treated by hemiablation with LDI ≥ 1. The treatment was well tolerated. Two phase III studies will reach completion in early 2015.

## Introduction

Currently, men who are diagnosed with early-stage prostate cancer face a difficult decision on their initial treatment. The current treatment choice for localized PCa lies between active surveillance and radical therapies [1, 2]. However, whole gland treatment may be unnecessarily aggressive [3–5], while active surveillance may not be suitable in a subset of patients with higher-risk disease [6]. Radical treatments of the prostate, such as radiotherapy and surgery, are associated with an important burden of side effects and long-term toxicities [3], and their use in early-stage prostate cancer is linked to overtreatment. For a subset of prostate cancer patients, there is a clear need for an alternative approach and interest has been renewed in focal therapy [7], which aims at selective ablation of the tumour and local control without the comorbidities frequently associated with whole gland treatment.

TOOKAD^®^ Soluble vascular-targeted photodynamic (VTP) therapy is a minimally invasive technique used as a focal therapy for patients with early prostate cancer. Its mode of action depends on three essential components: a light-sensitive drug to enhance the sensitivity of tumour vasculature to light energy; light of a specific wavelength; and sufficient oxygen to drive the reaction. Once optical fibres within hollow plastic needles are accurately positioned in the prostate, light of a specific wavelength is delivered through these fibres and activates an intravenously administered photosensitizer in the prostate. This generates reactive oxygen species (ROS) that activate thrombosis within the blood vessels. This photodynamic reaction results in obliteration of the vessel anatomy with resultant deprivation of oxygen and nutrients to the tumour cells and surrounding prostate tissue in the treated area.

TOOKAD^®^ Soluble (WST11, padeliporfin; palladium bacteriopheophorbide monolysotaurine) is a novel photosensitizers of the bacteriochlorophyll derivatives family. Unlike the previous compound (WST09) which needed solubilizing excipient Cremophor^®^ [8, 9], TOOKAD^®^ Soluble is soluble in aqueous solutions. TOOKAD^®^ Soluble presents minimal extravasation from the circulation and therefore remains confined to the vasculature even at high doses; it is rapidly cleared by hepatic and renal systems [10, 11]. Preclinical studies in several animal models have shown that treatment with TOOKAD^®^ Soluble leads to occlusion of the entire tumour vasculature within a few minutes of treatment, leading to tumour ablation [12–15].

A recently published retrospective analysis of 28 patients, treated with TOOKAD^®^ Soluble in multicentre trials [16], proposed a semi-empirical model based on three parameters of photodynamic therapy: dose of drug, energy fluence, and delivery time. This analysis was able to establish a correlation between the illuminated volume of prostate tissue and the necrosis as measured on day 7 magnetic resonance imaging (MRI) scan. Subsequently, three pilot studies of focal treatment with TOOKAD^®^ Soluble in patients with localized prostate cancer established that the parameters for the optimal photosensitizer and light combination to give maximum ablation of malignant prostatic tissue were 4 mg/kg TOOKAD^®^ Soluble activated by 753-nm light at a dose of 200 J/cm [17, 18]. The present paper reports the findings of a pooled analysis of these three studies. It was designed to more reliably evaluate the efficacy and safety of the recommended study drug and light dosage combination.

## Methods

### Studies and patients included in the meta-analysis

The three studies included in the pooled analysis (PCM201, PCM202, and PCM203) all looked at the efficacy of various combinations of light energy and TOOKAD^®^ Soluble doses (Table 1). Having established that TOOKAD^®^ Soluble at the dose of 4 mg/kg combined with a light energy of 200 J/cm at 730 nm provided the optimal conditions of treatment, in terms of efficacy and safety, only patients dosed with this combination were included in the pooled analysis. Patients who did not receive any amount of TOOKAD^®^ Soluble, or who received other doses of TOOKAD^®^ Soluble or other light energies were excluded.

**Table 1:** Study design of the three studies included in the pooled analysis

Study number	CLIN801 PCM201
ClinTrials.gov identifiers	NCT00707356/
EudrAct or IND Number	2008-000876-26
Number of patients enroled	42
Number of patients treated at optimal dose	35
Study design	Multicentre, phase II, open-label, single IV dose, 6-month clinical trial
TOOKAD^®^ Soluble treatment	Single, 10 min, IV administration, doses of 2 to 6 mg/kg, 753 nm laser light at a fixed power (150 mW/cm) and energy (200 J/cm)
Study number	CLIN901 PCM202/
ClinTrials.gov identifiers	NCT00946881/
EudrAct or IND Number	IND 101,886
Number of patients enroled	30
Number of patients treated at optimal dose	21
Study design	Prospective, multicentre, phase I/II, nonrandomized, open-label, single IV, escalating drug dose and light energy dosage clinical trial
TOOKAD^®^ Soluble treatment	Single, 10 min, IV administration, doses of either 2, 4 or 6 mg/kg, 753-nm laser light at escalating fixed energy doses of 200 J/cm and 300 J/cm, by escalating power at each energy to 167 mW/cm and 250 mW/cm, respectively
Study number	CLIN902 PCM203/
ClinTrials.gov identifiers	NCT00975429/
EudrAct or IND Number	2009-012809-19
Number of patients enroled	86
Number of patients treated at optimal dose	61
Study design	Multicentre, phase II, open-label, single IV dose, 6-month, nonrandomized clinical trial
TOOKAD^®^ Soluble treatment	Part A: patients were assigned to treatment groups based on the prostate size (<60 cc received 4 mg/kg WST11 and ≥60 cc received 6 mg/kg WST11). 200 J/cm light. Part B, patients were assigned to one of two treatment groups based on predefined criteria and received either 4 or 6 mg/kg WST11 and 200 or 300 J/cm light

### Inclusion criteria for the individual studies

The main inclusion and exclusion criteria for the individual studies were the same as reported in PCM201 [17] and PCM203 studies [18]. Dynamic contrast MRI was performed in all patients. PSA level had to be <10 ng/mL at entry. In PCM201 and PCM203, for men diagnosed by transperineal template guided biopsy, secondary Gleason pattern 4 was permitted provided that it was low burden (<3 cores positive per lobe and ≤3 mm maximum cancer core length). No restrictions were placed on the number of positive cores present at baseline in PCM201 or PCM203. In PCM202, ≤50 % of sampled cores were to be positive, each positive core having a tumour length of ≤5 mm, and only patients with unilateral disease at entry were included.

All the studies were conducted according to Good Clinical Practice (CPMP/ICH) regulations and the Declaration of Helsinki and subsequent amendments, and the protocols were approved by the ethics committees or institutional review boards at all participating centres. All patients gave their written informed consent to participate before any study-related activities were performed.

### TOOKAD^®^ Soluble *VTP procedure*

The details of the procedure have already been described in previous publications [17–20]. Regarding the population analysed TOOKAD^®^ Soluble treatment comprised a single IV administration of TOOKAD^®^ Soluble (STEBA Biotech, L–2613 Luxembourg) at a dose of 4 mg/kg, followed by local illumination of the targeted zone using a using 753-nm laser light at a fixed power (150 mW/cm) and energy (200 J/cm) delivered through transperineal interstitial optical fibres positioned in the prostate. These fibres were inserted under ultrasound guidance according to a treatment plan based on the MRI and the prostate biopsies. The treatment consisted in a hemiablation (treatment of only one lobe of the prostate) for patients with unilateral disease at entry or in a conservative subtotal ablation (treatment of both lobes) in case of bilateral disease. The total duration of the whole procedure was about 2 h (including anaesthesia, fibre placement, and illumination with the laser light).

A urinary catheter was left in situ until the next morning. The patient was required to wear protective eyewear and stay in low level light for the 1 h after the treatment.

For all three trials, if the month 6 biopsy was negative, the patient was followed up per local standard of care. In case of positive month 6 biopsy, a retreatment with TOOKAD^®^ Soluble VTP was proposed. The pooled analyses were performed on the three trials when all patients reached their month 6 biopsy assessment. All analyses were based on individual patient data. The analysis of month 12 data and of retreatment data was not included in the pooled analysis.

### Assessments

Efficacy of the treatment was assessed by a 12-core prostate biopsy, taken 6 months after TOOKAD^®^ Soluble procedure. Success was defined as a negative biopsy in the treated lobe(s) that initially contained the tumour. The secondary efficacy criteria were (1) change from baseline in PSA levels at 1, 3, and 6 months and (2) the MRI result on day 7 and 6 months.

Adverse events (AEs) were reported, laboratory evaluations were performed, and vital signs were measured periodically during the 6-month follow-up period; AEs were coded using the Medical Dictionary of Regulatory Activities, version 12.0 (PCM201) and 13.0 (PCM202 and PCM203). AE severity was graded according to the National Cancer Institute’s Common Terminology Criteria for AEs (CTCAE), version 4.0. The relationship of the AEs was assessed with respect to both the study treatment and the technical procedure. Urinary and erectile functions at baseline, and at 1, 3, and 6 months post-treatment, were assessed using the International Prostate Symptom Score (IPSS) and International Index of Erectile Function (IIEF-5) quality-of-life questionnaires, respectively.

### Data sets analysed

Three patient populations were defined: the efficacy population included all patients who received the TOOKAD^®^ Soluble procedure irrespective of any protocol deviation. The per protocol (PP) population included all patients from the efficacy population who received the entire procedure and had no major protocol deviations. The safety population included all patients who received the procedure. Efficacy presentations were produced on the efficacy population and repeated on the patients from the PP population with LDI ≥ 1. The LDI was defined as the ratio between total light-emitting length of inserted illuminating fibres (cm) and the baseline volume by planimetry (cm^3^) of targeted prostate (half the prostate volume for unilateral treatment and the whole prostate volume for bilateral treatment).

### Statistical methodology

Data were analysed using SAS (Version 9.1.3) and presented using descriptive statistics, with 95 % confidence intervals (CIs) also being provided for the percentage of patients with a negative biopsy assessment and for the mean change from baseline in PSA values. If a patient discontinued the study in the 3-6 month period following the Day 1 visit, a biopsy was performed at the time of discontinuation. These biopsy results were analysed with the other Month 6 biopsies.

Missing values were not replaced (unless specified otherwise) during the statistical analysis of safety or efficacy. No imputations were required for partial/missing dates for AEs. Baseline value was defined as the latest value prior to the first TOOKAD^®^ Soluble treatment. When several measurements were available at baseline, i.e. at screening or day 1 before anaesthesia, only the last data before the first treatment were used.

## Results

### Patient populations

A total of 117 men (mean age, 62.2 years) completed the entire TOOKAD^®^ Soluble procedure at the recommended dose and were included in the efficacy and safety sets (35, 21, and 61 men from studies PCM201, PCM202, and PCM203, respectively); 109 men were included in the PP set. Two patients discontinued prior to month 6, one patient due to lack of efficacy and one patient withdrew his consent. There were 38 men and 79 men, respectively, in the LDI < 1 and LDI ≥ 1 subgroups (Table 2).

**Table 2:** Patient disposition: overall, by study, and by LDI and laterality of treatment

	All studies	By study			By LDI and laterality			
		PCM201	PCM202	PCM203	LDI < 1		LDI ≥ 1	
					4 mg/kg 200 J/cm	4 mg/kg 200 J/cm	4 mg/kg 200 J/cm	4 mg/kg 200 J/cm
					Unilateral	Bilateral	Unilateral	Bilateral
	*N* = 117	*N* = 35	*N* = 21	*N* = 61	*N* = 20	*N* = 18	*N* = 68	*N* = 11
Efficacy/safety analysis sets	117	35	21	61	20	18	68	11
Per protocol set	109	29	21	59	18	15	65	11
Discontinued prior to month 6^a^	2 (1.7)	1 (2.9)	0	1 (1.6)	1 (5.0)	0	1 (1.5)	0
Reason for discontinuation	*n* = 2	*n* = 1	*n* = 0	*n* = 1	*n* = 1	*n* = 0	*n* = 1	*n* = 0
Withdrew consent	1 (50.0)	0	0	1 (100)	0	0	1 (100)	0
Lack of efficacy	1 (50.0)	1 (100)	0	0	1 (100)	0	0	0

*LDI* light density index

^a^Percentages are based on the number of patients included in the safety population

All patients had at least one positive core at baseline. The mean [standard deviation (SD)] time since the baseline biopsy was 4.7 (2.3) months, and the mean number of positive scores was 2.0 (range, 1–10). Almost all patients (116 of 117, 99.1 %) had a Gleason score of 3+3. Demographic and baseline characteristics were generally well balanced across the studies.

### Efficacy

#### Month 6 biopsy

In the efficacy population, month 6 biopsies were available for 114 patients and 142 treated lobes. By month 6, 78 out of 114 patients (68.4 %) had negative biopsies (95 % CI 59.1, 76.8 %) (Table 3). When the results were compared by LDI and laterality, men treated unilaterally with an LDI ≥ 1 fared the best, with 80.6 % of patients having a negative biopsy at month 6 (Table 3).

**Table 3:** Prostate biopsy results at month 6: overall and by LDI and laterality

	All studies	By study			By LDI and laterality			
		PCM201	PCM202	PCM203	LDI < 1		LDI ≥ 1	
					4 mg/kg 200 J/cm	4 mg/kg 200 J/cm	4 mg/kg 200 J/cm	4 mg/kg 200 J/cm
					Unilateral	Bilateral	Unilateral	Bilateral
	*N* = 117	*N* = 35	*N* = 21	*N* = 61	*N* = 20	*N* = 18	*N* = 68	*N* = 11
Prostate biopsy at month 6	114	34	21	59	19	17	67	11
Number (%) patients								
Positive biopsy	36 (31.6)	16 (47.1)	8 (38.1)	12 (20.3)	11 (57.9)	8 (47.1)	13 (19.4)	4 (36.4)
Negative biopsy	78 (68.4)	18 (52.9)	13 (61.9)	47 (79.7)	8 (42.1)	9 (52.9)	54 (80.6)	7 (63.6)
Exact 95 % CI^a^	(59.1, 76.8)	(35.1, 70.2)	(38.4, 81.9)	(67.2, 89.0)	(20.3, 66.5)	(27.8, 77.0)	(69.1, 89.2)	(30.8, 89.1)

If a patient discontinued the study prior to month 6 but more than 3 months following day 1, a biopsy was performed at the time of patient discontinuation. These biopsy results were included with the others

^a^For the percentage of patients with negative biopsy assessment

When the analyses were limited to the PP population treated with an LDI ≥ 1 (*N* = 76), the negative biopsy rate at month 6 was 77.6 % (95 % CI 66.6, 86.4 %) (Table 5), which was higher than seen in the overall efficacy population (68.4 %).

#### Day 7 MRI and PSA results

The mean percentage of necrosis of the targeted prostate tissue was defined by the volume of necrosis at day 7 in the treated lobes or lobe divided by the mean of the pre- and post-treatment of the whole prostate volume or half the prostate volume, respectively. This derived volume was used as the prostate tends to swell after the treatment, resulting in a larger prostate volume at day 7 than at baseline. Based on the MRI data, the mean percentage of necrotic prostate tissue on day 7 was 76.5 % (Table 4). Radiological evidence of extraprostatic necrosis was reported in about two-third of the patients (67.2 %), and this was usually not associated with clinically significant disorders. Mean changes from baseline in PSA levels were −2.1 and −2.0 ng/mL, respectively, at 3 and 6 months (Table 4). The PSA results should be interpreted with caution as the treatment given in this study is not a whole gland treatment.

**Table 4:** Post-treatment MRI Characteristics and PSA levels (efficacy population)

	Studies			All studies
	PCM201	PCM202	PCM203	
	*N* = 35	*N* = 21	*N* = 61	*N* = 117
Day 7 prostate MRI results, mean (SD)				
Prostate volume by planimetry (mL)	62.7 (24.2)	56.7 (23.4)	58.9 (18.4)	59.6 (21.1)
Volume of necrosis by planimetry (mL)	19.5 (8.9)	18.4 (10.9)	26.0 (13.5)	23.0 (12.5)
Percentage necrosis (%)	68.8 (34.5)	64.1 (25.3)	83.8 (20.9)	76.5 (26.6)
PSA levels (ng/mL), mean (SD)				
Baseline	5.6 (2.1)	3.7 (2.4)	6.2 (2.4)	5.6 (2.5)
At 1 month	4.5 (3.1)	4.7 (4.0)	4.9 (3.8)	4.8 (3.6)
Change from baseline at 1 month	−1.1 (3.6)	0.9 (3.1)	−1.2 (4.2)	−0.8 (3.9)
At 3 months	3.1 (1.8)	3.5 (3.4)	3.6 (2.2)	3.4 (2.3)
Change from baseline at 3 months	−2.6 (2.4)	−0.1 (2.6)	−2.5 (2.4)	−2.1 (2.6)
At 6 months	3.5 (3.2)	2.9 (2.2)	3.8 (2.5)	3.5 (2.7)
Change from baseline at 6 months	−2.1 (3.4)	−0.8 (1.0)	−2.4 (2.4)	−2.0 (2.7)

In the PP population with an LDI ≥ 1 (Table 5), the MRI scan at week 1 showed a mean percentage of necrotic tissue on day 7 of 86.3 %, with evidence of extraprostatic necrosis reported in approximately 77.3 % of patients. Mean changes from baseline in PSA levels were −2.3 ng/mL at both 3 and 6 months.

**Table 5:** Post-treatment MRI characteristics and PSA levels (PP population with LDI ≥ 1)

	Studies			All studies
	PCM201	PCM202	PCM203	
	*N* = 12	*N* = 15	*N* = 49	*N* = 76
Biopsy results at month 6				
Negative biopsy, *n* (%)	10 (83.3)	11 (73.3)	38 (77.6)	59 (77.6)
Exact 95 % CI	(51.6, 97.9)	(44.9, 92.2)	(63.4, 88.2)	(66.6, 86.4)
Day 7 MRI results, Mean (SD)				
Prostate volume by planimetry (mL)	46.9 (9.3)	62.3 (19.4)	56.0 (18.6)	55.8 (18.0)
Volume of necrosis by planimetry (mL)	17.9 (4.5)	21.5 (8.1)	24.3 (11.4)	23.0 (10.4)
Percentage necrosis (%)	96.4 (25.0)	74.3 (15.3)	88.5 (18.6)	86.3 (19.6)
PSA (ng/mL) results, mean (SD)				
Baseline	5.8 (2.8)	4.0 (2.1)	6.1 (2.5)	5.6 (2.6)
At 1 month	3.3 (3.2)	5.5 (4.4)	4.6 (2.9)	4.5 (3.3)
Change from baseline at 1 month	**−**2.4 (3.6)	1.3 (3.6)	**−**1.5 (3.3)	**−**1.1 (3.6)
At 3 months	2.6 (2.5)	3.8 (3.6)	3.4 (1.9)	3.3 (2.4)
Change from baseline at 3 months	−3.2 (3.4)	−0.1 (3.0)	−2.7 (2.2)	−2.3 (2.8)
At 6 months	1.9 (1.7)	2.8 (1.8)	3.8 (2.6)	3.3 (2.4)
Change from baseline at 6 months	−3.8 (2.7)	−1.2 (0.9)	−2.3 (2.4)	−2.3 (2.4)

### Treatment emergent adverse events

A total of 97 patients (82.9 %) reported 386 treatment emergent AEs (TEAEs) (Table 6). Treatment and procedure were well tolerated, and the majority of TEAEs were of mild or moderate intensity (CTCAE grades 1 or 2). Overall, 61 patients (52.1 %) reported at least 1 TEAE that was considered related to study drug, 53 patients (45.3 %) reported at least 1 TEAE that was considered related to the study device, and 90 patients (76.9 %) reported at least 1 TEAE that was considered related to the technical procedure. The most frequently reported TEAEs overall were dysuria (33.3 %), erectile dysfunction (16.2 %), perineal pain (15.4 %), haematuria (13.7 %), urinary retention (11.1 %), and micturition urgency (9.4 %); the majority of these commonly reported TEAEs were considered related to study drug, devices, or technical procedures of the study. No significant change in vital signs was observed.

**Table 6:** Summary of SAEs by NCI CTCAE toxicity grade, system organ class, and preferred term

System organ class/adverse event	Severity (NCI CTCAE v4.0 toxicity grade)					
	Gr1	Gr2	Gr3	Gr4	Gr5	Total
All system organ class	2	7	2	0	0	11
Gastrointestinal disorders	0	1	0	0	0	1
Haematemesis, oesophageal and duodenal ulcer	0	1	0	0	0	1
General disorders and administration site conditions	0	2	0	0	0	2
Necrosis	0	2	0	0	0	2
Infections and infestations	0	0	1	0	0	1
Orchitis	0	0	1	0	0	1
Renal and urinary disorders	0	2	1	0	0	3
Urethral stenosis	0	0	1	0	0	1
Dysuria	0	1	0	0	0	1
Haematuria	0	1	0	0	0	1
Reproductive system and breast disorders	2	1	0	0	0	3
Pelvic pain	1	0	0	0	0	1
Prostatitis	1	1	0	0	0	2
Vascular disorders	0	1	0	0	0	1
Deep vein thrombosis	0	1	0	0	0	1

No TEAEs led to study discontinuation or premature withdrawal from the study. Eleven patients (9.4 %) reported 11 serious TEAEs. Eight of them were considered as related to either study drug, study device or study procedure, and three were not related. Among the eight-related serious TEAEs, there were two cases of “large extraprostatic necrosis” reportedly due to a laser failure and two cases of “prostatitis”. All other related serious TEAEs were single occurrences (pelvic pain, haematuria, prostatic urethra stricture and worsening of orchi-epididymitis).

There were no notable changes in the haematological and biochemical parameters post-procedure, and the worst grades reported for most laboratory parameters were CTCAE grade 1 or 2. There was no evidence of renal toxicity.

### Quality-of-life questionnaires

**Fig. 1:**
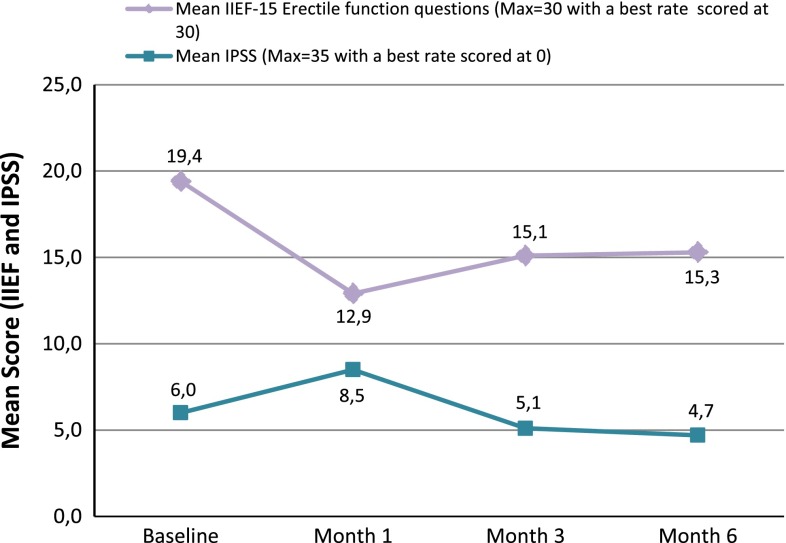
Mean IIEF-5 and IPSS (Questions 1–7) scores for PP patients with LDI ≥ 1

Urinary and erectile functions were assessed with the IPSS and IIEF-5 (Fig. 1). For both measures, there were slight reductions in the sum of scores at 6 months post-treatment. The mean (SD) change from baseline at month 6 was −1.8 (4.8) and −3.0 (7.0), for the IPSS and IIEF-5, respectively, indicating a slight improvement in urinary function and a slight deterioration in sexual function compared with baseline.

## Discussion

Focal treatment with TOOKAD^®^ Soluble may offer the potential to treat patients with early prostate cancer in a minimally invasive, targeted manner. It is a single session treatment, lending itself to administration in an ambulatory care setting and may be repeatable. The operative time of this technique is also one of the shortest of the anti-tumour, anti-vascular treatment modalities currently available [21, 22]. Because of the focal nature of delivery, and with TOOKAD^®^ Soluble photo activation and ROS generation confined within the circulation, rather than being aimed at cellular components, damage to surrounding tissue is kept to a minimum. Thus, there is a reduction in the significant morbidity (e.g. urinary incontinence and erectile dysfunction) associated with radical therapies. Three prospective studies that investigated the optimal treatment conditions for TOOKAD^®^ Soluble in early prostate cancer allowed to establish the recommended treatment conditions for prostate cancer tumour ablation as 4 mg/kg TOOKAD^®^ Soluble activated by 753-nm light at a dose of 200 J/cm and a light density index of >1. The present pooled analysis of the biopsy, MRI, PSA, and safety data from 117 patients who were treated with those optimal treatment parameters in these three studies gives a better evaluation of the safety and efficacy of this dose/light combination, which can achieve prostate ablation in the majority of patients. Of the patients in the pooled analysis who had received the recommended treatment combination, 68.4 % had negative biopsy results at month 6. For these patients, the necrosis percentage was 76.5 % at day 7. When these analyses were confined to patients in the PP set who had been treated with an LDI ≥ 1, the month 6 negative biopsy rate improved to 77.6 % and the necrosis percentage to 86.3 % at day 7. These results confirm results from the exploratory analysis in the individual studies that efficacy was improved when the LDI ≥ 1.

Overall, the tolerability and safety of TOOKAD^®^ Soluble were considered satisfactory. The majority of TEAEs were mild or moderate and no patient who received the complete TOOKAD^®^ Soluble procedure discontinued due to adverse effects. The most common TEAEs were dysuria, erectile dysfunction, and perineal pain. No significant modification in vital signs was reported. There were eight SAEs in eight patients considered to be related to the study procedure, all of them resolved without sequelae. A part from two necrosis events caused by excessive illumination due to device malfunction, most of the related SAEs were due to the insertion of the treatment needles and resolved in the days following the procedure without sequelae. For study PCM201 and PCM202, safety data were regularly reviewed by an independent Data and Safety Monitoring Board which did not identify any particular safety issue.

Even though most of the patients had no significant urinary symptoms at baseline, a reduction was seen in the IPSS questionnaire scores suggesting an improvement of urinary symptoms at month 6. The urinary catheter was successfully removed in the morning of the day following the procedure in the majority of patients treated. The results of the IIEF suggested a mild deterioration of the sexual quality of life of the patients. Post-procedural erectile dysfunction was reported as an AE in 16.2 % of patients following the TOOKAD^®^ Soluble procedure, which compares favourably with a reported incidence of 30–80.0 % following radical treatments [20]. However, these results are difficult to analyse since many patients were not sexually active, and a longer follow-up would be needed to assess the real impact of the procedure on erectile function as erectile dysfunction tends to improve with time after a surgical intervention.

### Limitations to the pooled analysis

The studies contributing to the meta-analysis were carried out in men who had low-risk prostate cancer, which might be predominantly considered suitable for active surveillance. Controversy continues to surround the need for intervention in early-stage prostate cancer, and a recent publication of a randomised control of surgery versus observation (PIVOT) concluded that conservative management may be the most appropriate approach for such men [4]. However, as around two-thirds of men in the UK and a higher proportion in Europe currently receive treatment for low-risk prostate cancer, it seems a reasonable patient population in which to explore the potential of a new treatment modality [18]. In order to further clarify a place for focal therapy, investigation in men with intermediate risk disease will be required.

Due to the limited sensitivity of TRUS biopsy, a negative biopsy at 6 months post-procedure does not necessarily confirm the absence of tumour. In the context of low-risk prostate cancer on standard TRUS biopsy, one in four men may have no cancer found on repeat TRUS, although one in four may be upgraded or upstaged [21]. We did consider using transperineal template guided biopsy both prior to and following the procedure, but decided that the requirement for an additional two general anaesthesia during a 6-month study would be too great a burden for the patient.

This pooled analysis looked at outcomes based on a short follow-up of 6 months, and longer term histological results and patient reported outcomes are needed. Meanwhile, the time-scale does allow an assessment of the short- to medium-term toxicity profile of this procedure, which is sufficient to show the ability of TOOKAD^®^ Soluble to reliably eradicate malignant lesions.

## Conclusions

This aim of this analysis of pooled data from three phases II studies was to evaluate further the efficacy and safety of a single dose of the recommended study drug and light dosage combination of TOOKAD^®^ Soluble in the focal treatment of patients with localized prostate cancer, 6 months after treatment. Negative biopsies were obtained in the majority of men at month 6. This negative biopsy rate improved further to 80.6 % in the 67 patients treated by hemiablation with LDI ≥ 1. The treatment was well tolerated. Further work to assess this within a European phase III randomized controlled trial comparing TOOKAD^®^ Soluble to active surveillance (NCT01310894) is ongoing, with recruitment of more than 400 men completed, as well as in a further phase III study in Latin America (NCT01875393).
